# Biomechanical analysis of conventional and sumo deadlift

**DOI:** 10.3389/fbioe.2025.1597209

**Published:** 2025-05-27

**Authors:** N. C. Hanen, K. Ben Mansour, G. N. Ertel, Y. Duchene, G. C. Gauchard

**Affiliations:** ^1^ Université de Lorraine, UR 3450 DevAH, Nancy, France; ^2^ Université de Lorraine, CARE, Nancy, France; ^3^ Université de Lorraine, Faculty of Sport Sciences, Villers-lès-Nancy, France

**Keywords:** biomechanics, dynamic, EMG, joint, powerlifting, strength, SPM

## Abstract

**Introduction:**

The conventional (CDL) and sumo (SDL) deadlifts are two fundamental techniques used in competitive lifting and as effective exercises for strengthening the knee and hip muscles. This study aims to investigate their biomechanical differences through a comprehensive analysis of joint kinematics, joint kinetics, and muscle activation.

**Materials and Methods:**

Thirty experienced male lifters performed both CDL and SDL at 85% of their one repetition maximum (1-RM). Lower limb joint range of motion (ROM), internal joint moments, and muscle activation of key lower limb and spinal muscles were recorded and analyzed. Paired t-tests and Statistical parametric mapping (SPM) were used to compare parameters between lifting techniques (p < 0.025).

**Results:**

SDL showed greater ROM in the frontal and transverse planes, particularly at the hip and knee, whereas CDL involved greater hip flexion and ankle dorsiflexion. CDL generated higher hip extension moments, while SDL produced greater frontal and transverse plane joint moments at the hip and knee. Additionally, SDL induced a greater ankle inversion moment. In the transverse plane, ankle moments were higher in CDL during phase 1 and became greater in SDL in phase 2. Regarding EMG peak values, the biceps femoris exhibited greater activation in CDL across both phases. The tibialis anterior and the erector spinae thoracis demonstrated greater activation in CDL during phase 1 and phase 2, respectively. Conversely, the vastus lateralis exhibited higher peak activation in SDL, but only during phase 1.

**Conclusion:**

CDL is more effective for targeting posterior chain, particularly the hip extensors, while SDL emphasizes anterior chain involvement and induces greater mediolateral stabilization demands. SDL may be particularly beneficial for knee reinforcement and increases frontal plane demands, supporting its relevance in rehabilitation contexts that require enhanced mediolateral stability. These findings highlight the importance of selecting the appropriate deadlift technique according to specific training or rehabilitation objectives.

## 1 Introduction

In modern athletic training, the barbell deadlift is a staple exercise routinely performed by athletes across a variety of disciplines such as rugby, weightlifting, American football, and others. This multi-joint, full-body exercise predominantly targets the posterior chain (Flandez et al., 2020), generating significant forces and neuromuscular activity, particularly in the lower limbs and the core (Bird and Barrington-Higgs, 2010; Choe et al., 2021). The deadlift plays a key role in developing both strength and power (Bird and Barrington-Higgs, 2010; Pratt et al., 2020).

The deadlift consists of three key events (McGuigan and Wilson, 1996; Hales et al., 2009; Jovanović et al., 2021). The first event, barbell lift-off, occurs when the athlete generates sufficient force to lift the barbell off the ground. The second event, knee passing, is defined as the instant when the barbell clears the knees. Finally, the third event, lift completion, is achieved when the athlete assumes a fully upright position with extended hips and knees, with scapular retraction (Hales et al., 2009). However, deadlift execution techniques can vary, with two primary styles forming the foundation for all other variations (Bird and Barrington-Higgs, 2010): the conventional deadlift (CDL) and the nonconventional deadlift. Among the nonconventional styles, the sumo deadlift (SDL) is the most commonly used by athletes and, along with CDL, is the only variation performed in competition. The main difference between the two techniques lies in hand placement. In the CDL, the hands are positioned outside the knees, while in the SDL, the hands are positioned inside the knees, accompanied by a wider, externally rotated foot stance. The SDL involves a shorter vertical displacement of the barbell and requires less mechanical work compared to the CDL (McGuigan and Wilson, 1996; Escamilla et al., 2000).

Despite the widespread use of deadlift, relatively few studies have focused on its biomechanical properties. Regarding kinematic aspects, studies comparing the two deadlift techniques have predominantly used 2D video analysis (Cholewicki et al., 1991; McGuigan and Wilson, 1996; Jovanović et al., 2021). In some cases, analyses were conducted using video recordings from two cameras, enabling the reconstruction of 3D coordinate data from 2D digitized images captured from each camera view (Escamilla et al., 2000). Results indicated that CDL practitioners exhibited a significantly greater average knee extension range during the barbell’s liftoff compared to SDL practitioners. Conversely, SDL practitioners were able to maintain a more upright posture at the start of the movement, with a significantly reduced trunk inclination angle, facilitated by a wider foot stance (Escamilla et al., 2000; Piper and Waller, 2001). Additionally, keeping the barbell closer to the body during the SDL reduces the lever arm stress, thereby decreasing mechanical stress on the lower back (Cholewicki et al., 1991; McGuigan and Wilson, 1996; Escamilla et al., 2000; Jovanović et al., 2021). Kinematic parameters computed using 2D video analysis and reconstructed 3D coordinate data from 2D digitized images were found to differ significantly, particularly for the SDL technique. In fact, Escamilla et al. (2000) reported significant discrepancies in all joint and segment angles during the SDL when comparing 2D and 3D analyses. This discrepancy was primarily attributed to the external rotation of the femur and a pronounced rotation of the feet outside the sagittal plane (Escamilla et al., 2000).

In terms of kinetic analysis, limited research has explored the kinetic variables associated with the two deadlift techniques. Without employing dynamometric sensors, Escamilla et al. (2000), Escamilla et al. (2001) calculated joint moments using quasi-static models and reported significant differences in ankle and knee moments between the CDL and SDL techniques, while hip moments were found to be comparable (Escamilla et al., 2000; 2001). However, the absence of dynamometric sensors in their analysis constitutes a critical limitation, potentially affecting the accuracy of the reported results. Other studies have investigated joint moments during deadlift tasks using force plates; however, they did not compare the CDL and SDL techniques. For example, Swinton et al. (2011) compared the use of a straight bar versus a hexagonal bar during CDL, while Lee et al. (2018) compared the CDL with the Romanian deadlift.

Regarding neuromuscular activations, few studies have compared these two techniques despite the extensive literature on deadlift variants (Martín-Fuentes et al., 2020). The literature presents conflicting findings. Escamilla et al. (2002) observed a significantly higher activation of the vastus lateralis and vastus medialis during SDL compared to CDL. However, Vitanza (2018) did not find any difference for the quadriceps and hamstring muscles. Similarly, concerning erector spinae activation, both Escamilla et al. (2002) and Vitanza (2018) reported no differences between techniques, whereas Cholewicki et al. (1991) found that spinal extension requirements are approximately 10% higher during CDL, leading to a significantly greater activation of the erector spinae.

These studies reveal inconsistent findings, emphasizing gaps in our understanding of the biomechanical implications of the two deadlift techniques. Moreover, the methodologies commonly used to examine the differences between the CDL and SDL techniques often suffer from notable technical limitations, especially those based on 2D measurements, highlighting the need for more rigorous and comprehensive research approaches to clarify these disparities.

Therefore, the aims of this study were to provide a biomechanical description and to investigate whether the CDL technique differs from the SDL technique and to assess these differences comprehensively. This comparison aims to provide valuable insights for developing training recommendations focused on injury prevention and performance optimization.

We hypothesized that the SDL technique would induce lower joint moments in the lower limbs, lower neuromuscular activation of the back and hip extensor muscles, and greater activation of the quadriceps during the movement execution.

## 2 Methods

### 2.1 Subjects

Thirty healthy and physically active men (age: 26 ± 4.6 years; weight: 81.3 ± 8.7 kg; height: 178.5 ± 6.2 cm) voluntarily participated in this study. *A priori* sample size calculations were performed using G*Power (version 3.1.9.4, University of Düsseldorf, Germany). The sample size of 29 participants was estimated based on an effect size of 0.7 to achieve a power of 95%, with alpha criterion of 0.05.

All participants were actively engaged in strength training and incorporated deadlift exercises into their routines at least once per week. They demonstrated a proficient execution of both CDL and SDL variations. The study was approved by the ethics committee of Comité de protection des personnes Sud Ouest et Outre Mer II (approval reference 2023-A02408-37) and was conducted in accordance with the requirements stipulated in the Declaration of Helsinki. All participants were informed about the procedures, purpose and possible risks associated with the experimental setup and gave their written consent prior to testing. Volunteers were required to have no musculoskeletal injuries or cardiovascular diseases within the past 6 months.

### 2.2 Experimental procedure

The experimental procedure consisted of three main phases: a standardized warm-up, an individualized 1-RM determination using an incremental loading protocol and a testing phase performed under submaximal load conditions set at 85% of the determined 1-RM.

#### 2.2.1 Warm-up and 1-RM adjustment protocol

Before starting the measurements, height and weight were collected from each participant. Participants were then equipped with reflective markers and neuromuscular surface electrodes. Subsequently, participants performed a standardized warm-up protocol consisting of 5 min of low-intensity exercise on an ergometer (Concept-2). This was followed by 10 repetitions of stiff-legged deadlifts, 10 repetitions of bent-over rows with a 20 kg Olympic barbell for each exercise, and 10 repetitions of deadlifts at 35% of their reported 1-RM.

Participants were asked to perform either the CDL or SDL deadlift barefoot using an ascending load protocol corresponding to 35%, 50%, 65%, and 75% of their reported 1-RM, in order to adjust their 1-RM to their athletic level of the measurement day. To control a potential effect of technique preference on the 1-RM estimation, the adjusted 1-RM was estimated for half of the population on their preferred technique, and for the other half on their non-preferred technique. Conventional deadlift was the favorite technique for 17 of the 30 participants. For the other half, their adjusted 1-RM was determined based on their non-preferential technique. Three repetitions were performed for each load condition in a continuous manner at highest speed during the ascending phase. This protocol allowed for the adjustment of the 1-RM estimation based on the force–velocity relationship, a method shown to be reliable and reproducible in deadlifts (Morán-Navarro et al., 2021). Between each load condition, participants had rest periods of 1 min 30 s, 2 min, 2 min 30 s, respectively. This progressive increase in rest time was intended to limit the effect of fatigue, adjusting the recovery periods according to the increasing loads lifted.

#### 2.2.2 Biomechanical analysis at 85% of 1-RM

Following the warm-up, 1-RM adjustment protocol and a 3 min recovery period, participants performed three repetitions at 85% of their adjusted 1-RM for each deadlift technique, in a randomized order. The selected 1-RM percentage ensured that the loads were sufficiently heavy to fall within the range for strength development (Peterson et al., 2004). Additionally, to ensure participant safety and control, the loads were kept below 90% of their 1-RM (Spencer and Croiss, 2015). Between each technique condition, participants had rest periods of 3 min. To ensure participants developed maximal power under each condition, they were instructed to perform the concentric phase of each repetition at maximum speed on each set. Between repetitions within a given technique, short pauses were imposed to ensure clear separation between the three repetitions at each load condition. The movement was performed with a “dead stop” to eliminate the effects of elastic energy.

**FIGURE 1:**
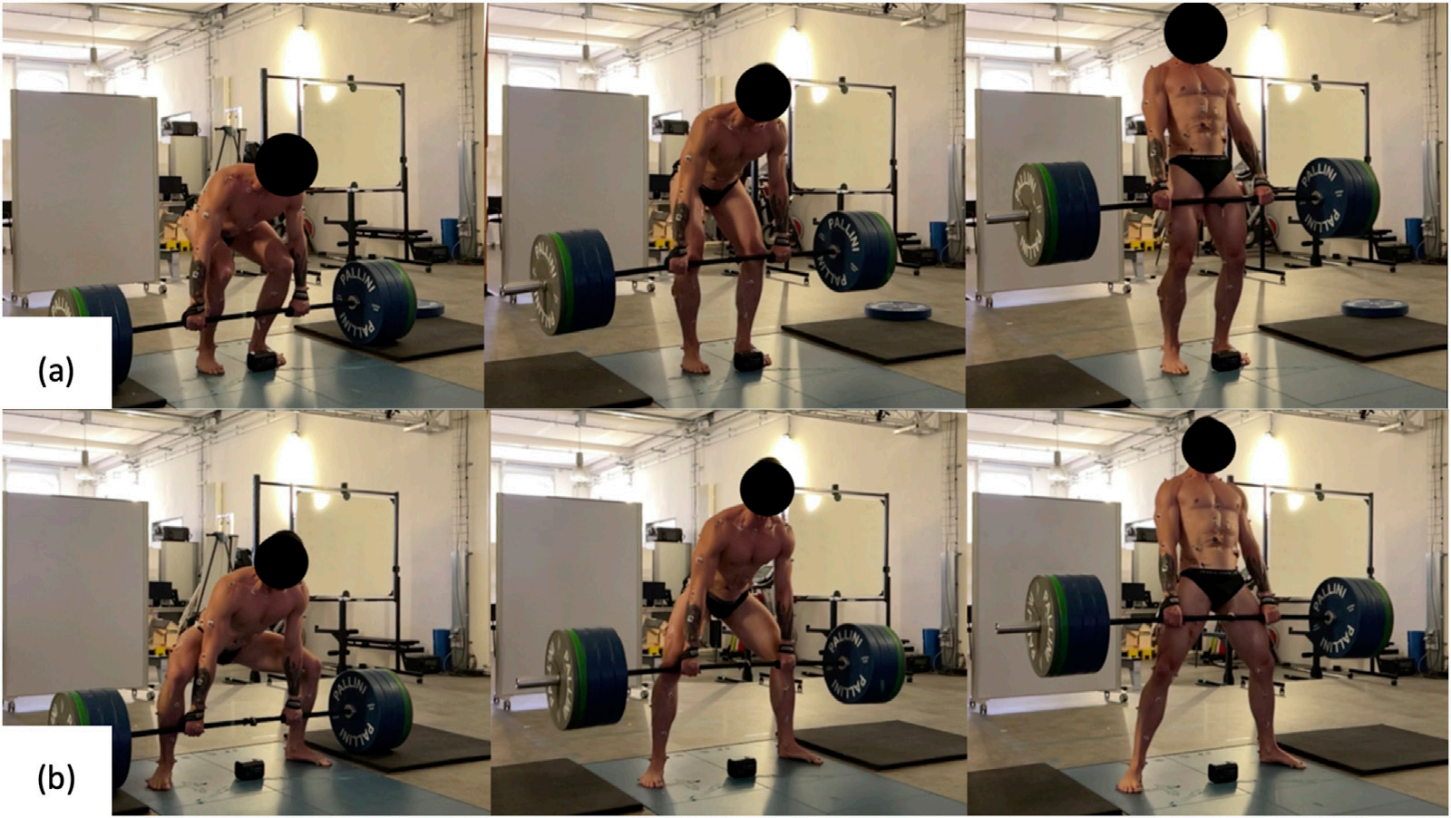
Conventional **(a)** and sumo **(b)** deadlift techniques at three key events: Lift-off; Knee passing and lift completion.

Regardless of the technique used, the movement began with the barbell resting on the ground. In the CDL, the feet were positioned in a narrow stance corresponding to the athlete’s natural width, with the hands gripping the barbell outside the knees and the hips positioned lower than the shoulders (Figure 1a). The barbell was lifted by simultaneously extending the hips and knees. In contrast, in the SDL, the main difference was the wider foot placement, with the toes pointed outward (Figure 1b). The hands gripped the barbell inside the knees. For both deadlift variations, an overhand grip was consistently used, although the grip width was adjusted according to the individual preferences of each participant. A trial was considered successful if, at the end of the concentric phase, the participant stood upright with fully extended knees and hips, a straight torso, and retracted shoulders.

### 2.3 Data collection

**FIGURE 2:**
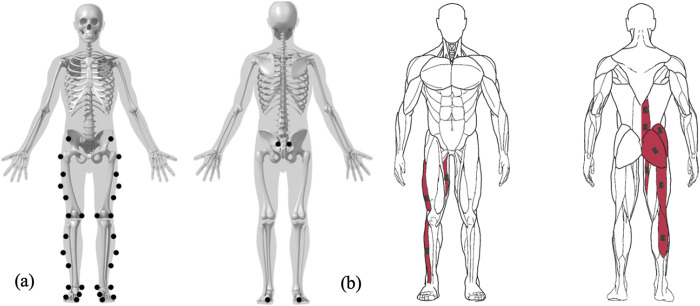
Marker **(a)** and EMG **(b)** placement on anatomical landmarks of the lower limbs.

Data collection included synchronized acquisition of ground reaction forces, 3D kinematics, and surface EMG signals. Ground reaction forces (GRF) in all three axes under each foot were measured using two triaxial force plates (BMS600900-2K, AMTI, USA) at a sampling frequency of 1000 Hz. Simultaneously, kinematic data were recorded at 200 Hz using a 14 cameras optoelectronic system (Arqus A5; QUALISYS Company, Sweden). A set of 34 reflective markers was placed on various anatomical landmarks of the participants’ lower limb (Figure 2a). Additionally, to track barbell height and identify the knee passing, three markers were positioned on the barbell: two at each end to track displacement, and one centrally to estimate vertical bar velocity.

To quantify neuromuscular activity, surface electromyography (EMG) data were recorded using nine Delsys Trigno EMG electrodes (Delsys, Natick, USA) at a sampling frequency of 2000 Hz. The skin was prepared by shaving, abrading, and cleaning to ensure optimal electrode contact. Electrodes were positioned according to the SENIAM recommendations on the following muscles: 1) vastus lateralis (VL); 2) biceps femoris (BF); 3) gastrocnemius lateralis (GL); 4) tibialis anterior (TA); 5) adductor magnus (AM); 6) gluteus maximus (GMax); 7) gluteus medius (GMed); 8) erector spinae at the L3 level (ESL); and 9) erector spinae at the T12 level (EST) (Figure 2b).

In the present study, since the deadlift involves symmetrical movement of the lower body segments (Escamilla et al., 2000; Salehi et al., 2020), kinematic, kinetic, and EMG data from the lower limbs were analyzed exclusively from the right side of the participants**.**

### 2.4 Data processing

The recorded data were segmented by repetition and then by phase. The first phase begins at barbell lift-off, defined as the moment when the barbell’s vertical height increases by 1% from its lowest position while resting on the floor, accounting for barbell deformation based on the vertical displacement of the barbell markers. At this instant, the athlete is already supporting the full weight of the barbell. The end of this phase is defined by knee passing, i.e., when the markers placed at the extremities of the barbell reach the vertical height of the femoral condyles during the movement. The initial tension of the barbell was not included in the analysis due to the variable degree of barbell deformation, which depends on the athlete’s grip width and the load. The second phase begins at knee passing and continues until the completion of the lift, defined as the instant when the lifter reaches an upright stance with fully extended knees and hips and retracted shoulders. In the present study, the knee passing event was selected for delineating the transition between movement phases, rather than the sticking point. Although the sticking point is of particular interest, given that success in weightlifting depends on the continuous upward displacement of the barbell through this mechanically challenging region, it was not observed in our data. The sticking point is typically characterized by a temporary decrease in barbell velocity, often occurring around the level of the inferior patella (Hales et al., 2009). However, due to the use of submaximal loading (85% 1-RM) in our protocol, no clear and identifiable sticking point nor identifiable sticking region were detected, as barbell velocity did not exhibit a marked deceleration prior to reacceleration. As noted by Kompf and Arandjelović (2016), the sticking point is neither an inevitable nor universal feature of resistance exercises. It usually emerges under conditions where a loss of mechanical advantage or intersegmental coordination causes a transient deceleration or failure point. Such conditions may not emerge at moderate intensities, where neuromuscular and mechanical constraints are insufficient to induce a distinct sticking region.

Marker trajectories, GRF, and EMG data were recorded synchronously using Qualisys Track Manager software (Qualisys AB, Gothenburg, Sweden). Marker trajectories and GRF data were low-pass filtered using a zero-lag, fourth-order Butterworth filter with cutoff frequencies of 12 Hz (Choe et al., 2021). EMG data were band-pass filtered using a zero-lag, fourth-order Butterworth filter, with cutoff frequencies of 30–450 Hz (Lu et al., 2018; Ertel et al., 2023). A low-pass filter with a cutoff of 6 Hz was applied to compute a linear envelope of the EMG signal, enabling muscle activity to be expressed as a percentage of maximum voluntary contraction (MVC). The filtered data were then exported to Visual3D software (C-Motion, Rockville, Maryland) for the computation of ankle, knee and hip joint angles and internal moments. For each phase, the data were then time-normalized to a scale of 0%–100%.

The peak EMG activation obtained during the task (i.e., maximal activation between both techniques) performed at a submaximal non-isometric voluntary contraction (i.e. 85% of 1-RM) was used as the normalization value (Burden, 2010; Lee et al., 2018).

### 2.5 Statistical analysis

The range of motion (ROM) of lower limb joints, mean peak values of joint moments, and EMG activity were assessed across repetitions and phases for each participant in both deadlift variations. Normality was assessed using a Shapiro-Wilk test, after which a paired Student’s t-test was performed to analyze the effect of the deadlift technique on ROM, joint moments, and EMG activity. Moreover, for each phase, a one-dimensional Statistical Parametric Mapping (SPM) analysis was conducted to allow a temporal comparison of the techniques, for each joint angle, internal joint moment and EMG signal across movement phases. After applying the Bonferroni correction for repeated statistical testing across the two phases, the significance level for all statistical tests was set at p < 0.025. All the statistical analyses were conducted using RStudio software (R Core Team, Vienna, Austria).

## 3 Results

### 3.1 Kinematics

#### 3.1.1 Range of motion

Focusing on the ROM of the lower limbs (Table 1), ankle angles showed significant differences between both techniques during phase 1 only. The SDL induced a greater ROM in the sagittal and frontal planes during phase 1 (p = 0.002), whereas a reduced ROM was observed in the transverse plane (p < 0.001). The CDL exhibited greater dorsiflexion during phase 1 (p = 0.005).

**TABLE 1:** Range of motion (mean ± SD [min max]) for the ankle, knee and hip during conventional and sumo deadlifts across phases 1 and 2 in all three anatomical planes. Bolded values indicate significant differences (p < 0.025) between techniques within the same phase, as determined by paired t-tests.

Kinematics		Phase 1				Phase 2			
		Conventional	Sumo	P Value	Cohen’s d	Conventional	Sumo	P Value	Cohen’s d
Ankle Range (°)	Dorsiflexion (+) Plantar flexion (−)	**12,8 ± 4,0 [-1,1 11,7]**	**15,0 ± 3,3 [−7,8 7,3]**	**0.005**	−0,56	5,2 ± 1,9 [−2,9 2,1]	6,0 ± 1,9 [−12,0 −6,0]	0.030	−0,42
	Eversion (+) Inversion (−)	**3,2 ± 1,2 [-3,7 −0,5]**	**4,3 ± 2,1 [6,4 10,6]**	**0.002**	−0,61	4,1 ± 2,1 [−1,1 2,9]	5,2 ± 2,6 [10,4 15,7]	0.035	−0,40
	Internal rotation (+) External rotation (−)	**6,2 ± 2,0 [-10,6 −4,4]**	**4,5 ± 2,0[−7,9 −3,5]**	**< 0.001**	0,80	5,3 ± 2,8 [−5,6 −0,4]	5,9 ± 3,0 [−5,3 0,7]	0.179	−0,25
Knee Range (°)	Extension (+) Flexion (−)	**33,4 ± 6,9 [-58,6 −25,2]**	**38,1 ± 8,3 [−59,1 −20,9]**	**0.004**	−0,58	19,4 ± 5,6 [−25,7 −6,5]	19,1 ± 5,4 [−22,0 −2,9]	0.777	0,05
	Adduction (+) Abduction (−)	**4,4 ± 1,8 [-1,8 2,6]**	**7,0 ± 3,8 [1,8 8,8]**	**< 0.001**	−0,69	3,6 ± 2,0 [−1,1 2,5]	4,8 ± 2,5 [−1,2 3,6]	0.044	−0,39
	Internal rotation (+) External rotation (−)	5,5 **±** 2,2 [6,0 11,5]	5,7 ± 2,7 [3,6 9,3]	0.797	−0,05	6,5 ± 3,1 [5,6 11,9]	6,4 ± 2,6 [3,7 10,1]	0.865	0,03
Hip Range (°)	Flexion (+) Extension (−)	**38,3 ± 6,1 [53,3 91,7]**	**39,9 ± 5,8 [46,6 86,5]**	**0.006**	−0,54	**49,4 ± 9,7 [3,7 53,3]**	**43,9 ± 11,2 [2,7 46,6]**	**< 0.001**	0,96
	Adduction (+) Abduction (−)	**3,0 ± 1,0 [−8,4 −5,3]**	**7,9 ± 4,1 [−31,6 −23,6]**	**< 0.001**	−1,10	**3,8 ± 1,5 [−9,1 −5,4]**	**5,1 ± 2,1 [−24,6 −19,6]**	**0.020**	−0,45
	Internal rotation (+) External rotation (−)	**8,9 ± 3,9 [−11,8 −2,9]**	**15,4 ± 3,7 [−20,7 −5,4]**	**< 0.001**	−1,95	**6,0 ± 2,0 [−15,8 −9,8]**	**10,1 ± 3,6 [−30,1 −19,9]**	**< 0.001**	−1,03

For the knee joint, SDL resulted in a greater ROM in the sagittal (p = 0.004) and frontal planes (p < 0.001) during phase 1, while no difference was observed in phase 2.

At the hip joint, SDL led to an increased ROM in all three planes during phase 1 (p = 0.006 for frontal plane; p < 0.001 for sagittal and transverse plane), as well as greater ROM in the frontal (p < 0.001) and transverse planes (p < 0.001) during phase 2. However, CDL exhibited a greater hip extension ROM during phase 2 (p = 0.025).

#### 3.1.2 Joint angles: SPM analysis

**FIGURE 3:**
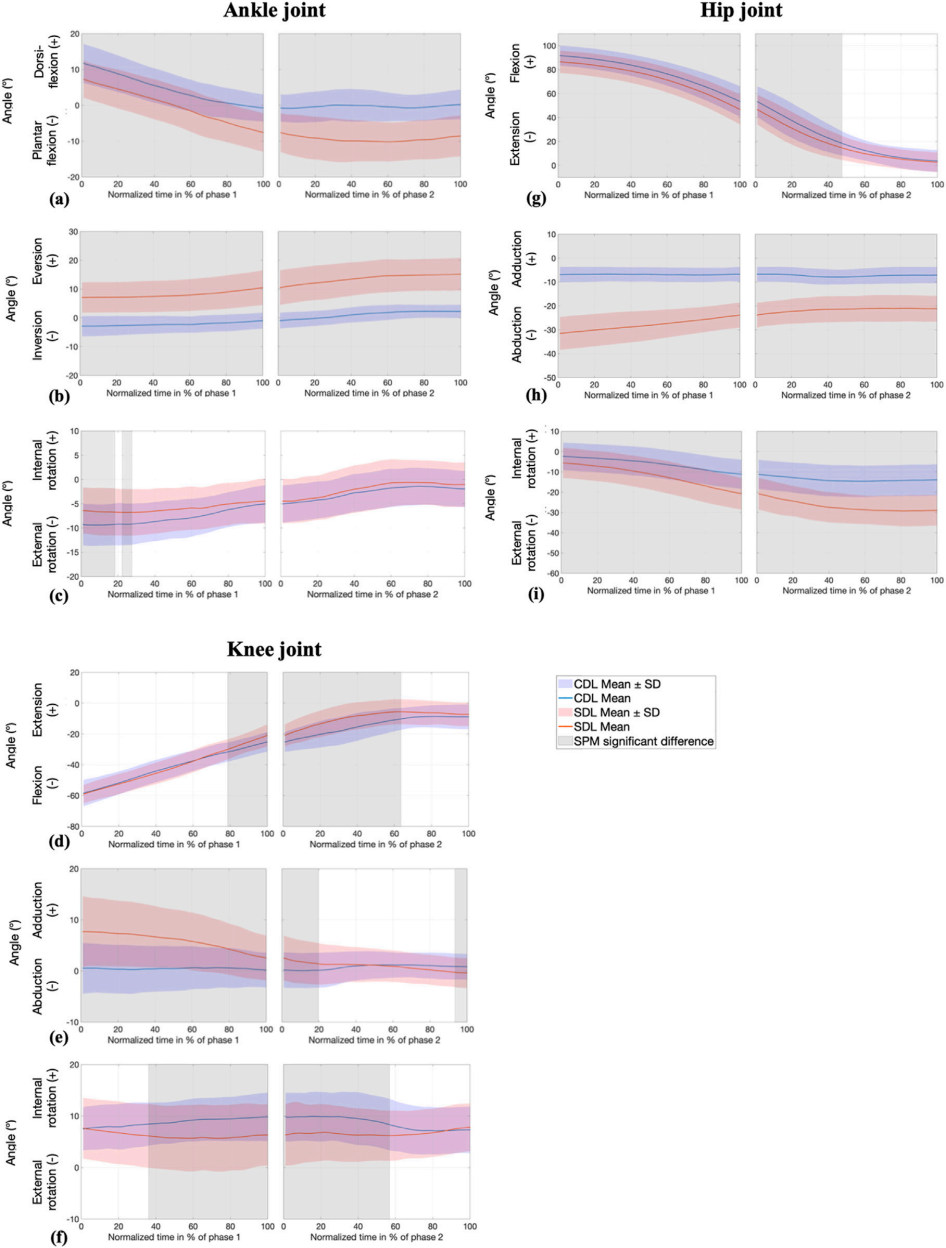
SPM analysis of joint angles (°) at the ankle (sagittal **(a)**, frontal **(b)**, and transverse **(c)** planes) knee (sagittal **(d)**, frontal **(e)**, and transverse **(f)** planes), and hip (sagittal **(g)**, frontal **(h)**, and transverse **(i)** planes) during conventional (CDL) and sumo (SDL) deadlifts across phases 1 and 2. Blue and red lines represent the CDL and the SDL deadlifts. Grey areas indicate significant differences (p < 0.025) between techniques.

SPM analysis showed a significant difference for ankle, knee, and hip angles between techniques and across phases and planes, as illustrated in Figure 3, which indicates the specific time intervals during which these differences reach statistical significance. Specifically, CDL resulted in greater ankle dorsiflexion from the initiation of the movement, stabilizing around a neutral position, whereas SDL induced plantarflexion starting at 53% of the first phase and continuing until the full completion of the lift. In SDL, the ankle exhibited eversion throughout the entire movement. The conventional stance allowed the ankle to maintain a lower angulation in the frontal plane, transitioning from inversion to eversion at the beginning of the second phase. However, the ankle was significantly more externally rotated in CDL compared to SDL during the first part of phase 1, approximately until 27% of this phase.

The angle of knee flexion differed significantly, mainly around knee passing, from 79% of the first phase to 63% of the second phase, with an earlier extension observed in SDL. Additionally, the knee in SDL exhibited an initial adduction of approximately 8°, which gradually decreased throughout the movement. In contrast, CDL maintained a nearly neutral frontal plane angle. The difference between the two techniques persisted during the first quarter of the second phase.

Hip angles exhibited significant variations throughout the entire movement, both in the frontal and transverse planes. Specifically, hip abduction and external rotation were greater in SDL compared to CDL, while hip flexion was more pronounced in CDL until the midpoint of the second phase.

### 3.2 Kinetics

#### 3.2.1 Peak joint moments

As shown in Table 2, analysis of the peak lower limbs joint moments using paired t-tests revealed significant differences between techniques across movement phases (p < 0.025). In CDL, an ankle eversion moment was observed in both phases, whereas SDL was characterized by an inversion moment. An external rotation moment at the ankle was greater in CDL during phase 1 before reversing in phase 2 (p < 0.001). Knee moments were larger for SDL on the two phases (p < 0.001), except in the frontal plane during phase 2 where no difference was observed. Hip extension moments were higher in CDL, whereas in frontal and transverse plane hip moments were greater in SDL (p < 0.001).

**TABLE 2:** Peak joint moments (mean ± SD) for the ankle, knee and hip during conventional and sumo deadlifts across phases 1 and 2 in all three anatomical planes. Bolded values indicate significant differences (p < 0.025) between techniques within the same phase, as determined by paired t-tests.

Kinetics		Phase 1				Phase 2			
		Conventional	Sumo	P Value	Cohen’s d	Conventional	Sumo	P Value	Cohen’s d
Ankle (Nm)	Dorsiflexion (+) Plantar flexion (−)	−122,2 ± 31,9	−120,4 ± 31,4	0.683	−0,08	−100,7 ± 30,3	−92,8 ± 31,1	0.085	−0,33
	Eversion (+) Inversion (−)	**29,6 ± 15,3**	**−10,8 ± 13,7**	**< 0.001**	2,48	**19,1 ± 12,6**	**−15,2 ± 9,8**	**< 0.001**	2,74
	Internal rotation (+) External rotation (−)	**−15,8 ± 8,6**	**−21,1 ± 10,5**	**0.005**	0,56	**−18,2 ± 7,0**	**−10,5 ± 8,0**	**< 0.001**	−1,01
Knee (Nm)	Extension (+) Flexion (−)	**52,8 ± 30,4**	**80,2 ± 35,7**	**< 0.001**	−0,94	**−59,4 ± 19,6**	**−75,9 ± 31,5**	**< 0.001**	0,74
	Adduction (+) Abduction (−)	**−32,6 ± 17,0**	**−68,9 ± 29,5**	**< 0.001**	1,48	−36,8 ± 14,9	−38,2 ± 28,7	0.721	0,07
	Internal rotation (+) External rotation (−)	**15,2 ± 14,9**	**42,1 ± 22,9**	**< 0.001**	−1,77	**−0,2 ± 8,1**	**10,3 ± 11,9**	**< 0.001**	−1,15
Hip (Nm)	Flexion (+) Extension (−)	**−303,3 ± 47,0**	**−276,2 ± 47,1**	**< 0.001**	−1,47	**−219,3 ± 40,5**	**−195,8 ± 41,7**	**< 0.001**	−1,24
	Adduction (+) Abduction (−)	**30,2 ± 16,1**	**101,0 ± 53,3**	**< 0.001**	−1,51	**−47,7 ± 23,7**	**65,5 ± 36,5**	**< 0.001**	−2,38
	Internal rotation (+) External rotation (−)	**−39,9 ± 21,5**	**−57,7 ± 26,5**	**< 0.001**	1,04	**−37,4 ± 12,1**	**−44,7 ± 18,3**	**0.001**	0,66

#### 3.2.2 Joint moments: SPM analysis

**FIGURE 4:**
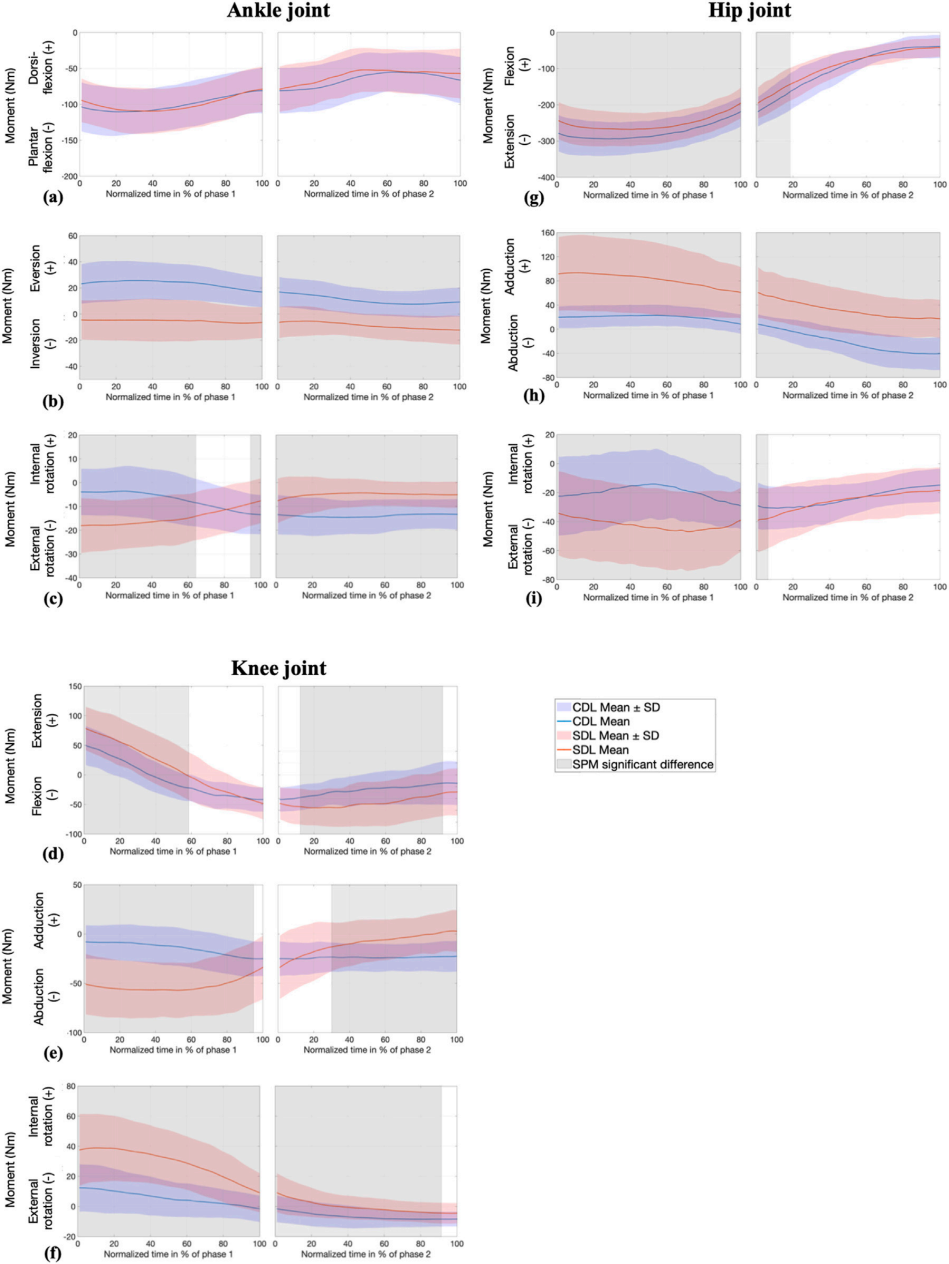
SPM analysis of joint moments (Nm) at the ankle (sagittal **(a)**, frontal **(b)**, and transverse **(c)** planes) knee (sagittal **(d)**, frontal **(e)**, and transverse **(f)** planes), and hip (sagittal **(g)**, frontal **(h)**, and transverse **(i)** planes) during conventional (CDL) and sumo (SDL) deadlifts across phases 1 and 2. Blue and red lines represent the CDL and the SDL deadlifts. Grey areas indicate significant differences (p < 0.025) between techniques.

Contrary to the sagittal plane, significant differences in ankle joint moments were observed in the frontal and transverse planes, as shown in Figure 4. The ankle exhibited an eversion moment during the CDL, whereas the SDL generated an inversion moment (less than 10 Nm) throughout the entire movement, with the peak moment occurring in the first phase. In the transverse plane, as the bar approached knee passing (the end of phase 1), the ankle’s external rotation moment increased in the CDL and decreased in the SDL. Specifically, up to 62% of phase 1, the external ankle moment was more pronounced in SDL, while in CDL, it became more accentuated during the last 4% of phase 1 and remained dominant throughout phase 2.

Knee joint moments were significantly different across all three planes. The knee extension moment was greater in SDL from 0% to 59% of phase 1. Subsequently, as the bar approached knee passing, the moment shifted from extension to flexion, with the decline occurring more sharply in SDL compared to CDL. In the frontal plane, SDL induced a greater knee abduction moment, which gradually decreased until no significant differences were observed between the two techniques in the final portion of phase 1 and until the first 30% of phase 2. From this point onward, SDL generated a knee adduction moment, while CDL maintained a stable knee abduction moment. Throughout the entire movement, an internal rotation moment was observed at the knee, with a greater magnitude in SDL, despite a progressive decrease.

Regarding the hip joint, the extension moment was greater in CDL during phase 1 and the first 19% of phase 2. The hip adduction moment was significantly greater in SDL throughout the entire movement. In contrast, CDL initially produced a hip adduction moment, which then transitioned to an abduction moment starting from 16% of phase 2 onward. Regarding the transverse plane, both techniques generated an external rotation moment at the hip, which was initially more pronounced in SDL during phase 1 before decreasing and showing no significant differences between the techniques from 7% of phase 2 onward.

### 3.3 EMG

#### 3.3.1 EMG peak value

As presented in Table 3, analysis of peak EMG activity using Wilcoxon signed-rank tests revealed significant differences between techniques for BF, EST, GMed, TA and VL. BF exhibited greater activation in CDL across both phases (p = 0.009 and p = 0.003 respectively). EST showed a significant difference in phase 2, with higher activation during CDL (Med: 74.8%, IQR: 12.8) compared to SDL (Med: 67.1%, IQR: 18.3).

**TABLE 3:** Normalized EMG activity (%MVC) in conventional and sumo deadlifts across phases 1 and 2. Bolded values indicate significant differences (p < 0.025) between techniques within the same phase as determined by Wilcoxon signed-rank tests.

EMG	Phase 1				Phase 2			
	Conventional	Sumo	P Value	Rank-biserial Correlation	Conventional	Sumo	P Value	Rank-biserial Correlation
Adductor	77,5 (12,1)	72,3 (21,2)	0.070	0,394	50,8 (28,9)	48,4 (25,3)	0.115	0,345
Biceps Femoris	**78,0 (13,1)**	**71,3 (15,6)**	**0.009**	0,535	**77,6 (18,9)**	**69,9 (20,9)**	**0.003**	0,604
Erector Spinae Lumbar	77,2 (16,7)	74,7 (27,9)	0.400	0,190	76,5 (29,9)	67,0 (21,6)	0.400	0,190
Erector Spinae Thoracis	78,6 (23,9)	75,5 (16,9)	0.966	0,011	**74,8 (12,8)**	**67,1 (18,3)**	**0.022**	0,485
Gastrocnemius Lateralis	73,8 (16,1)	73,4 (15,1)	0.984	0,006	67,1 (15,6)	68,1 (21,0)	0.584	−0,118
Gluteus Maximus	79,1 (20,0)	71,2 (16,1)	0.465	0,157	77,2 (13,3)	74,2 (20,9)	0.177	0,286
Gluteus Medius	74,6 (18,3)	75,9 (19,6)	0.824	0,049	80,4 (9,1)	75,9 (23,5)	0.038	0,432
Tibialis	**81,9 (17,9)**	**64,3 (26,3)**	**< 0.001**	0,699	64,7 (18,4)	63,3 (22,0)	0.903	−0,028
Vastus Lateralis	**55,5 (20,3)**	**63,3 (23,1)**	**0.014**	−0,517	49,1 (19,8)	40,0 (21,3)	0.084	0,370

TA demonstrated greater activation in CDL during phase 1 (Med: 81.9%, IQR: 17.9) compared to SDL (Med: 64.3%, IQR: 26.3; p < 0.001), but no significant difference was observed in phase 2. Conversely, VL exhibited higher peak activation in SDL during phase 1 (Med: 63.3%, IQR: 23.1) compared to CDL (Med: 55.5%, IQR: 20.3; p = 0.014).

#### 3.3.2 EMG SPM analysis

**FIGURE 5:**
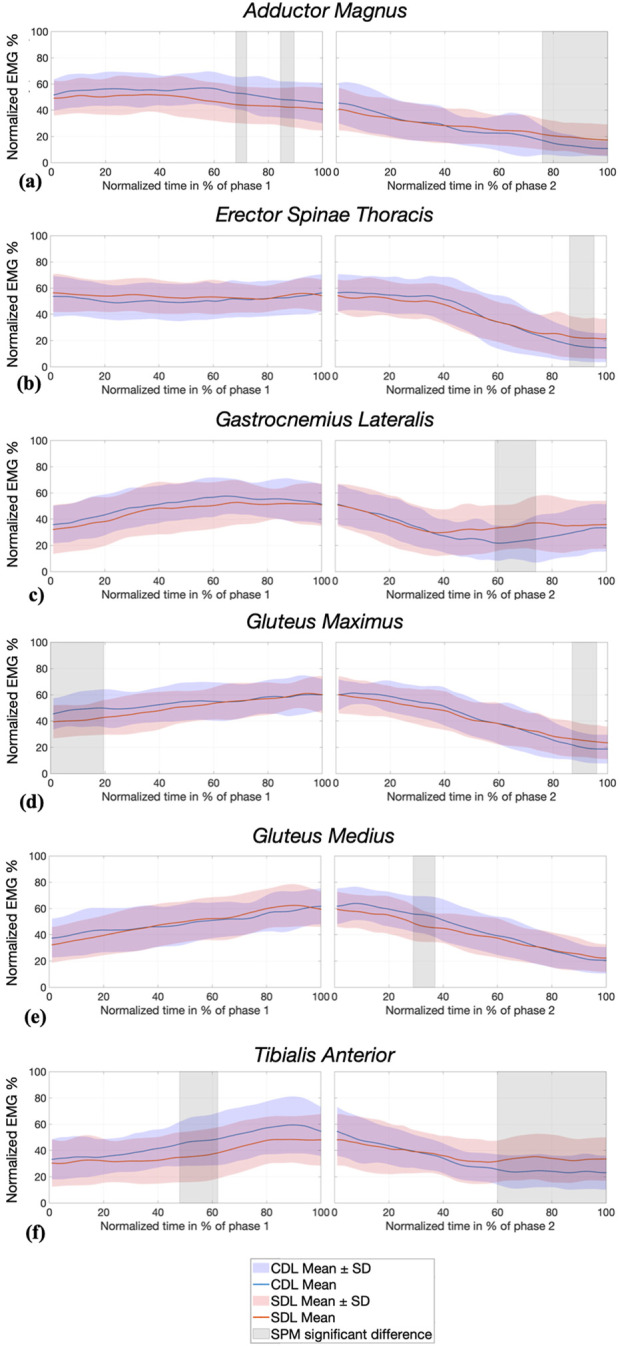
SPM analysis of normalized EMG activity (%) of AM: Adductor Magnus **(a)**, EST: Erector Spinae Thoracis **(b)**, GL: Gastrocnemius Lateralis **(c)**, GMax: Gluteus Maximus **(d)**, GMed: Gluteus Medius **(e)** and TA: Tibialis Anterior **(f)** during conventional (CDL) and sumo (SDL) deadlifts across phases 1 and 2. Blue and red lines represent the CDL and the SDL deadlifts. Grey areas indicate significant differences (p < 0.025) between techniques.

Significant differences were observed in muscle activation between the CDL and SDL techniques, as illustrated in Figure 5. The AM, EST, GL, and GMax muscles demonstrated higher activation during phase 2 of the SDL technique. More precisely, AM activation was significantly greater during the final quarter of the last phase (76%–100%), EST activity was greater in the last 10% (86.5%–95.5%) and the GL activation was greater between 59% and 74%.

Conversely, GMax and TA activation were significantly greater during phase 1 of the CDL technique (respectively 0%–19.5% and 48%–62%), and then greater during phase 2 of the SDL (respectively 87%–96% and 60%–100%). Additionally, GMed activation was significantly higher in CDL between 29% and 37% of phase 2, after knee passing.

## 4 Discussion

The objective of this study was to describe and analyze the conventional (CDL) and sumo (SDL) deadlift techniques through a comprehensive biomechanical analysis involving joint kinematics, internal joint moments, and muscle activation. The present findings highlight substantial differences in joint behavior between the two techniques across all three anatomical planes.

Three-dimensional lower limb kinematics revealed significant differences at the ankle and knee joints during phase 1, and at the hip joint throughout both phases. In the sagittal plane, SDL showed greater hip extension ROM during phase 1, despite CDL exhibiting the highest initial hip flexion at movement onset. Conversely, during phase 2, CDL demonstrated greater hip extension ROM. SPM analysis confirmed that significant differences between the two techniques extended from lift initiation to the midpoint of phase 2, with CDL consistently showing greater hip flexion. The increased hip flexion observed at the beginning of phase 2 in CDL, followed by a convergence of joint angles at full extension, underscores the greater overall hip ROM required by this technique. This pattern reflects the increased demand on hip mobility during this phase of the CDL.

Similarly, in the sagittal plane, SDL demonstrated greater knee extension ROM during phase 1. Although no significant difference was found in maximal flexion, SPM analysis revealed greater extension in SDL from the end of phase 1 through most of phase 2, indicating distinct inter-joint coordination strategies between the two lifting techniques. Differences in knee and hip angles between the two techniques progressively diminished as the lift approached completion. These sagittal plane findings are consistent with the existing literature. In fact, Jovanović et al. (2021) reported a greater knee extension ROM in SDL during phase 1 and greater hip extension ROM in CDL during phase 2. Similarly, Escamilla et al. (2000) analyzed only phase 1, from lift off to knee passing, and found greater hip and knee extension ROM in SDL.

At the ankle, sagittal plane ROM was significantly greater in SDL during phase 1. However, SPM analysis showed that CDL induced greater dorsiflexion throughout most of the lift. This distinction extended across both phases: CDL began with pronounced dorsiflexion trending toward neutral, while SDL transitioned into plantar flexion from mid-phase 1 to lockout. Although Escamilla et al. (2000) did not report ankle joint angles directly, their descriptions of initial posture and segment alignment suggest a similar mechanical configuration, characterized by a greater forward tibial inclination in CDL.

In the frontal and transverse planes, SDL demonstrated greater hip abduction, hip external rotation, and ankle eversion. Conversely, CDL involved greater ankle dorsiflexion and external ankle rotation, consistent with a movement strategy more heavily oriented toward the sagittal plane. Notably, hip external rotation in SDL increased progressively through the lift, while hip abduction decreased as the lower limbs extended. These patterns can be attributed to the initial setup: SDL is characterized by a wider stance and greater lower limb external rotation, which facilitates hip abduction and plantar flexion. In contrast, CDL adopts a narrower, sagittally aligned posture, with nearly parallel foot placement and more vertically oriented tibiae, thereby limiting plantarflexion and maintaining dorsiflexion throughout the lift. Although no significant difference in knee rotation ROM was observed, SPM analysis showed greater internal knee rotation in CDL from mid-phase 1 to mid-phase 2. Additionally, greater ROM in external ankle rotation was observed in CDL during phase 1, with SPM confirming these differences at movement onset. Escamilla et al. (2000) highlighted the limitations of 2D assessments in capturing out-of-sagittal-plane motion, particularly in SDL. To our knowledge, this is the first study to quantitatively characterize frontal and transverse plane joint behavior over time in both deadlift techniques. These findings underscore the importance of phases, temporal and three dimensional analyses in advancing the understanding of deadlift biomechanics, extending beyond the predominantly sagittal plane perspectives of previous studies (Cholewicki et al., 1991; McGuigan and Wilson, 1996; Escamilla et al., 2000; Jovanović et al., 2021).

The analysis of internal joint moments revealed further distinctions between the two techniques. Significant differences in hip joint moments were observed across all three anatomical planes throughout the movement. Notably, the application of SPM analysis provided valuable temporal insights that complemented peak value interpretations. In the sagittal plane, CDL produced greater hip extension moments during phase 1, which progressively diminished after the knee passing. Beyond this event, the difference between techniques became less pronounced, as both exhibited an increase in hip extension moment during phase 2, as highlighted by the SPM analysis. In the frontal plane, SDL was characterized by greater hip adduction moments early in the lift. Conversely, CDL demonstrated a progressive shift toward hip abduction moments as the lift progressed. In the transverse plane, SDL exhibited significantly greater hip external rotation moments during phase 1. These moments gradually decreased, and no significant differences between techniques were detected beyond the early part of phase 2. Overall, these results suggest that CDL emphasizes sagittal plane hip extension during the initial lifting phases, while SDL imposes greater mediolateral and rotational control demands at the hip joint.

Consistent with our initial hypothesis, SDL generated a significantly greater peak knee extension moment. However, SPM analysis showed that this difference declined as the barbell approached the knee passing event. After this instant, SDL exhibited a knee flexion moment instead of extension. This could be due to the bar being positioned anterior to the knees, a pattern that became less prominent around the lockout position at the end of the lift. In the frontal plane, SDL also generated more pronounced knee abduction moments, especially during phase 1, coinciding with peak frontal plane hip loading. These kinetic differences reflect the influence of stance width and limb orientation on load distribution in the frontal and sagittal planes, especially during phase 1, when joint moments were at their highest, as demonstrated by SPM analysis.

At the ankle, sagittal plane joint moments remained relatively consistent between techniques throughout both phases, as indicated by peak and SPM analyses. This contrasts with the findings of Escamilla et al. (2000), who reported a dorsiflexion moment during SDL and a plantarflexion moment during CDL. This discrepancy could be attributed to the load imposing a constant plantar flexion moment to achieve an upright position. A methodological limitation of their study is that joint moments were estimated solely from kinematic data, without incorporating ground reaction forces. Instead, moments were derived from segmental lever arms, which may have affected the accuracy of their kinetic estimations and could explain the differences observed with our findings (Escamilla et al., 2000). In the present study, both techniques exhibited a consistent plantarflexion moment, which may reflect the mechanical requirement to resist the barbell’s forward torque and maintain an upright posture. Although no significant differences in sagittal ankle joint moments were observed between conditions, previous research on wide stance squats (Larsen et al., 2021) has shown that wider stances are associated with greater laterally directed ground reaction forces, resulting in a more medially oriented net force vector. These adaptations may reflect a redistribution of joint loading in the frontal plane. These findings highlight distinct joint loading strategies between the two techniques: CDL predominantly relies on sagittal plane hip extension moments during early lift phases, while SDL generates greater frontal and transverse plane moments, particularly at the hip and knee joints. For instance, depending on the phase and focus of knee rehabilitation, conventional deadlifts performed at submaximal loads may represent a more controlled option before progressing to sumo variations that impose higher demands on frontal plane stabilization.

Regarding muscle activation, the SDL technique was hypothesized to involve greater recruitment of the quadriceps and adductor magnus, and lower activation of the back and hip extensor muscles. However, our EMG results only partially supported this expectation. Although SDL involved wider stance and higher frontal plane hip moments, peak activation of the adductor magnus did not differ significantly between techniques. Nonetheless, SPM analysis revealed greater activation in SDL towards the end of the movement in the sagittal plane, coinciding with the phase showing the lowest overall activation. This suggests that the adductor magnus may not have played a primary role in force generation during the lift. Conversely, peak value of the vastus lateralis during phase 1 was larger in SDL condition, aligning with our hypothesis that the anterior chain would be more activated in SDL. However, SPM analysis did not reveal any significant differences at specific time points, possibly due to anthropometric differences influencing individual recruitment strategies and increasing variability. The hypothesis that posterior chain muscles in CDL were more engaged during the ascending phase required further consideration. As for the posterior chain, the gluteus medius showed higher activation in CDL in the sagittal plane, consistent with the higher hip abduction moment observed in this technique. Peak activation values suggested no differences between the two techniques for the gluteus maximus. However, the SPM analysis indicated greater recruitment of the gluteus maximus at initiation of the lift in CDL, coinciding with the more flexed position and greater hip extension moments early in the movement. Furthermore, contrary to previous findings, no significant differences were observed during phase 1 for the erector spinae muscles. Yet, differences emerged during phase 2, supporting the hypothesis of increased recruitment in CDL. At the end of the lift, the SPM analysis of the gluteus maximus and the erector spinae thoracis revealed higher activation in SDL, though at significantly lower levels compared to peak values. While the gluteus maximus showed higher activation toward the end of the movement in SDL according to the SPM analysis, no significant difference was observed in peak values. The end of the movement corresponds to the lockout phase, marking the completion of the movement. A potential hyperextension at the hips and back could explain these differences, although this was not directly assessed in our study. In fact, Cholewicki et al. (1991) reported an increase in the back extension moment in CDL, and Salehi et al. (2020) observed increased erector spinae activity. Back muscle activation was expected to increase at lift initiation, as reported by Jovanović et al. (2021) who observed greater trunk inclination during phase 1. In contrast, our results did not align with these findings. The differences in participant characteristics and the weight lifted between our study and Jovanovic’s research may explain the contrasting patterns observed across the two studies. Regarding our population, one possible explanation is that the barbell may have moved further ahead of the knees, thereby increasing the back extension moment and the activity of the erector spinae in SDL. Analyzing the bar path would be valuable to validate this hypothesis. The increased activation of the adductor magnus, erector spinae thoracis, and gluteus maximus toward the end of the movement suggests that locking the bar required greater muscle recruitment to stabilize the position.

Overall, while some activation patterns support the idea of anterior chain emphasis in SDL and posterior chain emphasis in CDL, EMG findings were heterogeneous and often phase dependent. These results highlight the complexity of neuromuscular coordination in multi-joint movements like the deadlift and suggest that inter-individual variability and barbell mechanics may substantially influence muscle recruitment strategies.

Future research should extend beyond lower limb analysis to assess the mechanical constraints on the lumbar spine under training-relevant loads, enabling an estimation of spinal injury risk, which is a major concern in powerlifting. Additionally, incorporating participants' anthropometric characteristics into the analysis may provide valuable insights into how body segment ratios (legs, torso, arms) influence the studied variables. A deeper understanding of these biomechanical differences and their effects on load distribution and joint stress during the lift could help develop evidence-based recommendations for injury prevention.

## 5 Conclusion

This study highlights distinct biomechanical characteristics between conventional and sumo deadlift techniques. The conventional deadlift is associated with greater posterior chain involvement, particularly through increased hip extension demands and higher activation of the biceps femoris and thoracic erector spinae. In contrast, the sumo deadlift elicits greater activation of the vastus lateralis and produces higher joint moments not only in the sagittal plane at the knee but also in the frontal and transverse planes at both the hip and knee, reflecting greater anterior chain involvement and increased mediolateral stabilization demands. Given these differences, selecting the appropriate deadlift variation may help tailor strength training strategies according to muscular emphasis and joint loading profiles. The deadlift is a polyarticular movement that effectively develops the entire body, and its versatility allows modifications to suit specific development or rehabilitation objectives.

## Data Availability

The raw data supporting the conclusions of this article will be made available by the authors, without undue reservation.
